# Primary posterior mediastinal epithelioid angiosarcoma presenting as epigastric pain: long-term survival achieved via multimodal salvage therapy after early recurrence

**DOI:** 10.1186/s13019-026-04130-9

**Published:** 2026-04-24

**Authors:** Shouyong Xiao, Siyun Wu, Xianfeng Shao, Guangjian Li, Lianhua Ye

**Affiliations:** 1 Department of Thoracic Surgery I, The Third Affiliated Hospital of Kunming Medical University,Yunnan Cancer Hospital, Peking University Cancer Hospital Yunnan, Kunming, Yunnan China; 2 Health Examination Center, The Third Affiliated Hospital of Kunming Medical University,Yunnan Cancer Hospital, Peking University Cancer Hospital Yunnan, Kunming, Yunnan China

**Keywords:** Mediastinal tumor, Epithelioid angiosarcoma, Multimodal therapy, Intrapleural infusion, Case report

## Abstract

We report a rare case of primary posterior mediastinal epithelioid angiosarcoma (EA) in a 68-year-old male who presented with atypical upper abdominal pain. Contrast-enhanced chest computed tomography revealed a left posterior mediastinal mass, and histopathology with immunohistochemistry (CD31+, CD34+, FLI-1+) confirmed the diagnosis. Since preoperative imaging suggested a benign tumor, the patient underwent macroscopic complete resection via video-assisted thoracoscopic surgery (VATS). Histopathology with immunohistochemistry (CD31+, CD34+, FLI-1+, Ki-67 ~ 25%) unexpectedly confirmed the diagnosis of EA. However, within one month, he developed symptomatic hemorrhagic pleural effusion, and repeat exploration confirmed local recurrence with pleural metastasis. Given the aggressive nature of EA and early relapse, a multimodal salvage regimen was initiated, consisting of systemic paclitaxel (80 mg/m² on days 1, 8, 15 every 4 weeks, six cycles) and intrapleural cisplatin instillation (40 mg weekly for two doses). Remarkable clinical response was achieved, with resolution of pleural effusion and sustained disease control. The patient has remained alive with good quality of life for over 20 months post-recurrence. This case highlights that even in rapidly recurrent mediastinal EA, intensive multimodal therapy can achieve long-term survival. Our experience supports the integration of systemic chemotherapy with locoregional control strategies in managing aggressive sarcomas.

## Introduction

Epithelioid angiosarcoma (EA) is a highly aggressive subtype of vascular sarcoma characterized by epithelioid morphology and poor prognosis [1]. It most commonly arises in the skin of elderly patients, particularly on sun-exposed areas such as the head and neck [2]; however, visceral or deep-seated origins—including the liver, breast, and mediastinum—are associated with worse outcomes due to delayed diagnosis and limited treatment options [3].

Primary mediastinal EA is exceedingly rare, with fewer than 50 cases reported in the literature to date [4]. Due to its nonspecific symptoms—such as chest pain, dyspnea, or cough—it is often misdiagnosed initially, especially when the presentation mimics gastrointestinal or musculoskeletal disorders [5]. In this context, our patient presented with isolated upper abdominal discomfort, which delayed recognition of the true origin in the posterior mediastinum.

Surgical resection remains the cornerstone of curative intent for localized disease; however, recurrence rates exceed 70%, typically within the first year [6]. For recurrent or metastatic EA, there is no standardized chemotherapy regimen. Paclitaxel-based therapy has shown promising activity in soft tissue angiosarcomas, including epithelioid variants [7], while intracavitary cisplatin perfusion has been used palliatively for malignant effusions [8]. Nevertheless, the combination of systemic chemotherapy with regional control through intrapleural infusion has not been well documented in mediastinal EA.

Here, we present a case of early-relapsing primary posterior mediastinal EA successfully managed with a multimodal salvage approach, resulting in prolonged survival beyond 20 months, which compares favorably to the typically poor prognosis associated with this aggressive disease [9]. We also review current evidence and propose a rational strategy for high-risk cases.

## Case presentation

**Fig. 1:**
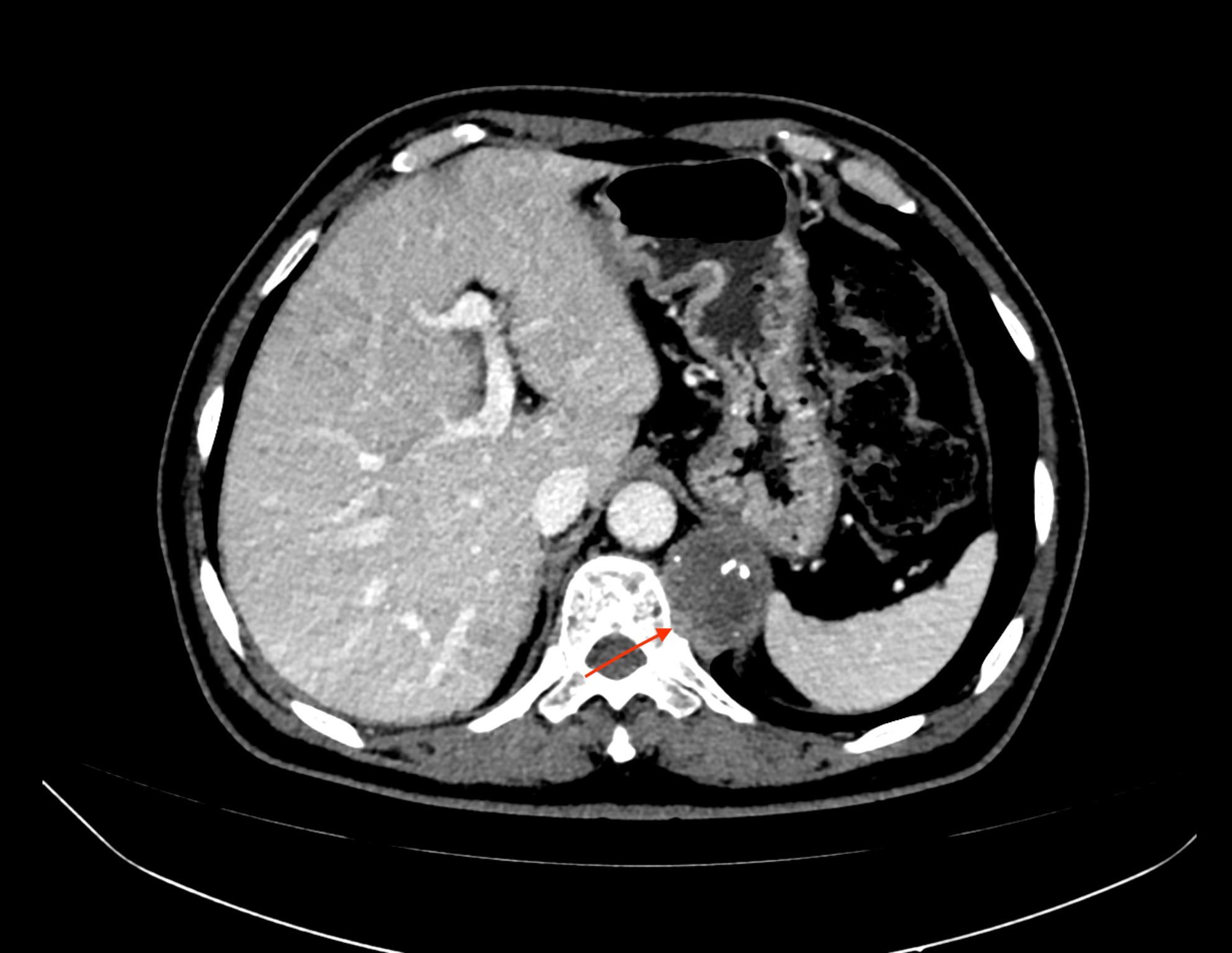
Preoperative chest computed tomography (CT). Contrast-enhanced CT imaging reveals a well-defined, heterogeneous mass measuring approximately 5.2 × 4.1 cm located in the left posterior mediastinum, adjacent to the thoracic vertebrae

A 68-year-old male presented with a two-week history of dull, non-specific epigastric pain and mild fatigue. He had no significant past medical history and denied weight loss, fever, or respiratory symptoms. Initial laboratory tests were unremarkable except for a slightly elevated D-dimer level. Contrast-enhanced chest computed tomography (CT) revealed a well-defined, heterogeneous mass measuring approximately 5.2 × 4.1 cm in the left posterior mediastinum, adjacent to the thoracic vertebrae and compressing the descending aorta (Fig. 1). No distant metastases or enlarged lymph nodes were identified.

**Fig. 2:**
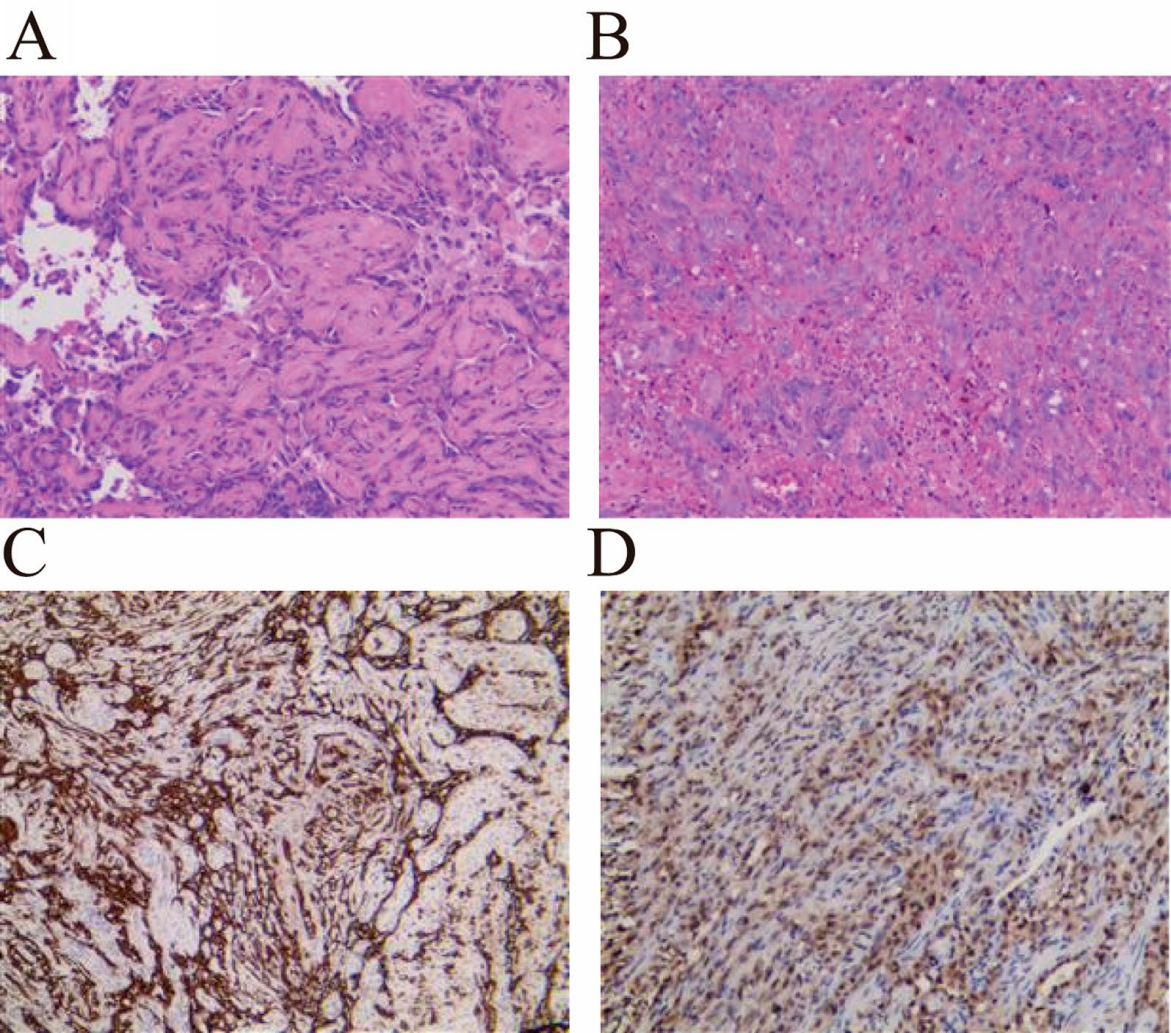
Histopathological and immunohistochemical characterization. (A) Hematoxylin and eosin (H&E) staining demonstrates sheets and nests of epithelioid cells characterized by abundant eosinophilic cytoplasm, vesicular nuclei, and frequent mitotic figures. (B–D) Immunohistochemical staining confirms the diagnosis of epithelioid angiosarcoma with diffuse positivity for (B) CD31, (C) CD34, and (D) FLI-1.

Since both in-house CT and external MRI strongly suggested a benign posterior mediastinal neurogenic tumor (e.g., schwannoma), a preoperative PET-CT scan was not routinely indicated. On August 17, 2023, the patient underwent video-assisted thoracoscopic surgery (VATS). Intraoperatively, the tumor was encapsulated, exhibited focal cystic changes with necrosis, and was dissected from surrounding structures with macroscopic clearance achieved. Final pathological examination demonstrated sheets and nests of epithelioid cells with abundant eosinophilic cytoplasm, vesicular nuclei, and frequent mitotic figures. Immunohistochemical staining showed diffuse positivity for CD31, CD34, and FLI-1 (Fig. 2A-D). Notably, the Ki-67 proliferative index was approximately 25%, indicating high mitotic activity. This unexpectedly confirmed the diagnosis of epithelioid angiosarcoma. Due to the initial clinical suspicion of a benign tumor, formal microscopic margin assessment (R0/R1) and pleural invasion status were unfortunately not delineated in the standard pathology report.

The patient recovered uneventfully and was discharged on postoperative day 5, before any standard adjuvant therapy could be planned due to the unexpected sarcoma diagnosis. However, just three weeks after surgery (September 7, 2023), he returned with worsening left-sided chest pain and progressive dyspnea. Repeat chest CT revealed a newly developed moderate left pleural effusion with hemorrhagic characteristics. A chest tube was inserted, yielding over 1100 mL/day of bloody fluid. Cytological analysis of the pleural fluid was negative for malignant cells, but serial hemoglobin levels showed progressive decline, which was directly attributed to massive, active intrathoracic bleeding.

**Fig. 3:**
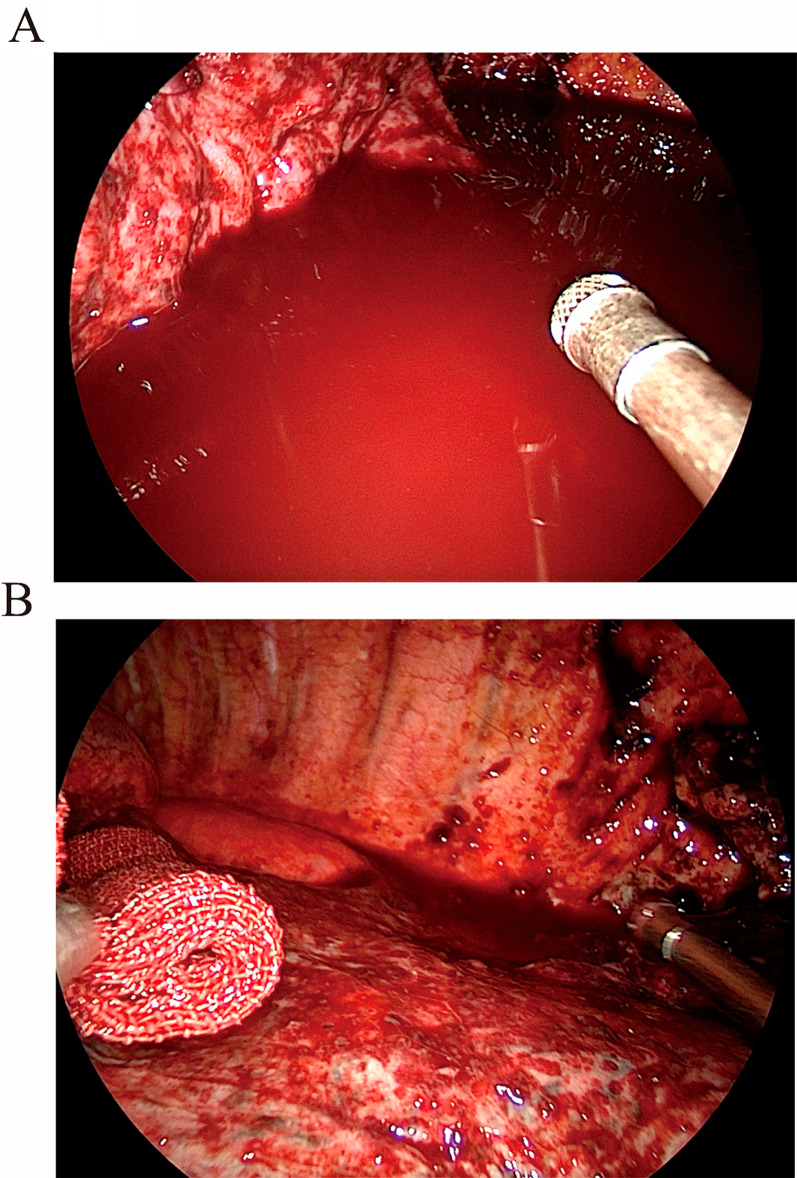
Intraoperative findings during emergency salvage thoracotomy. (**A**) Massive hemorrhagic pleural effusion observed upon entering the thoracic cavity. (**B**) Intraoperative view after suctioning the bloody effusion, revealing pleural dissemination

**Fig. 4:**
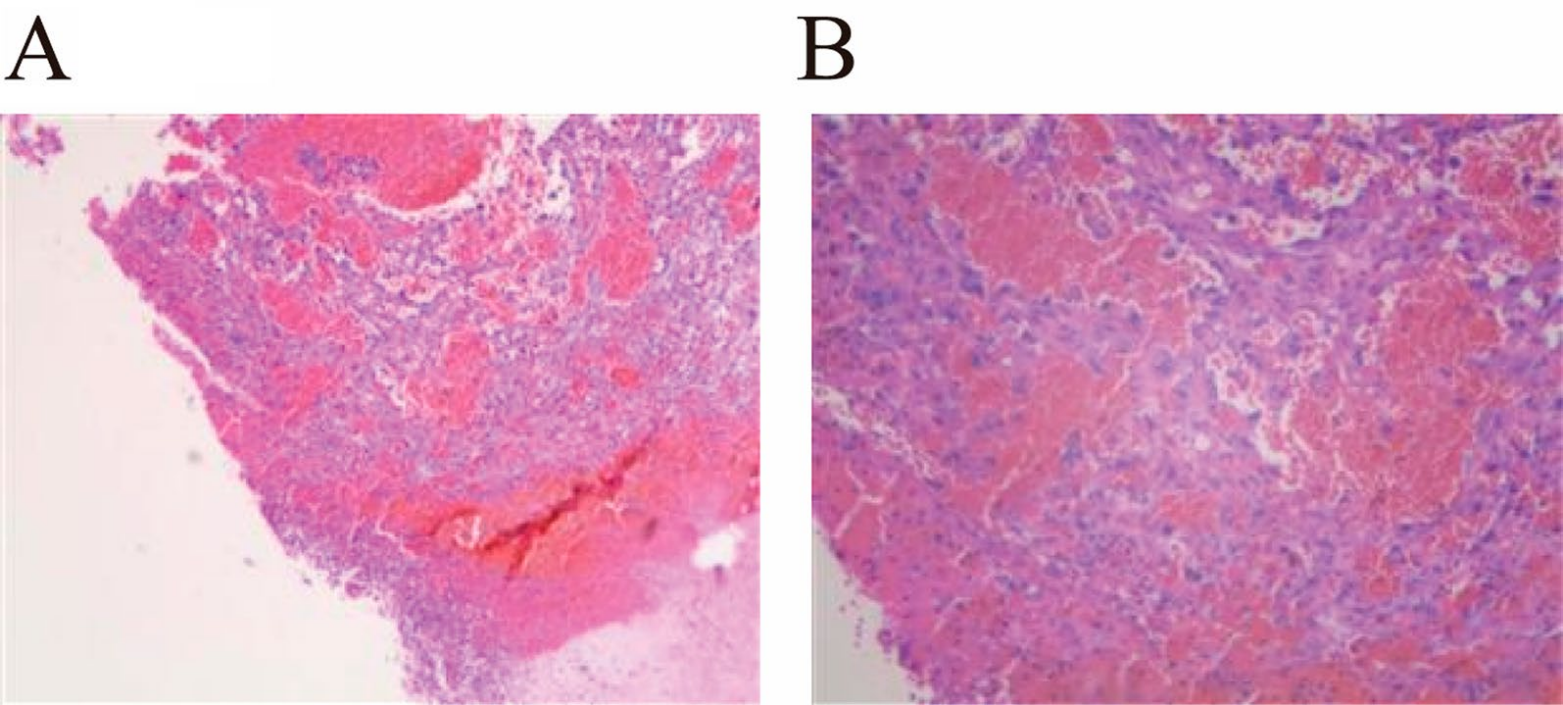
Pathological confirmation of early recurrence. (**A**-**B**) Hematoxylin and eosin (H&E) staining of the pleural specimens confirming the recurrence of epithelioid angiosarcoma, characterized by atypical cells and extensive hemorrhage

Due to this life-threatening clinical deterioration, an emergency salvage thoracotomy was performed on September 20, 2023, taking absolute clinical precedence over initiating systemic adjuvant therapy. Intraoperative findings included massive hemorrhagic pleural effusion and thickened parietal pleura near the previous surgical bed, highly suggestive of local recurrence with pleural dissemination (Fig. 3A–B). Pathological and cytological examinations of the pleural specimens confirmed recurrent epithelioid angiosarcoma(Fig. 4A–B). Extensive pleurectomy was performed, which involved stripping the parietal pleura near the surgical bed and ablating macroscopic visceral nodules to achieve maximal cytoreduction and hemostasis.

Given the aggressive early relapse, a multimodal salvage therapy strategy was initiated. The regimen consisted of systemic chemotherapy with paclitaxel (80 mg/m² intravenously on days 1, 8, and 15 every 4 weeks) for six cycles, combined with intrapleural cisplatin instillation (40 mg weekly for two doses) administered via the indwelling chest drain to target residual microscopic disease and control malignant effusion.

**Fig. 5:**
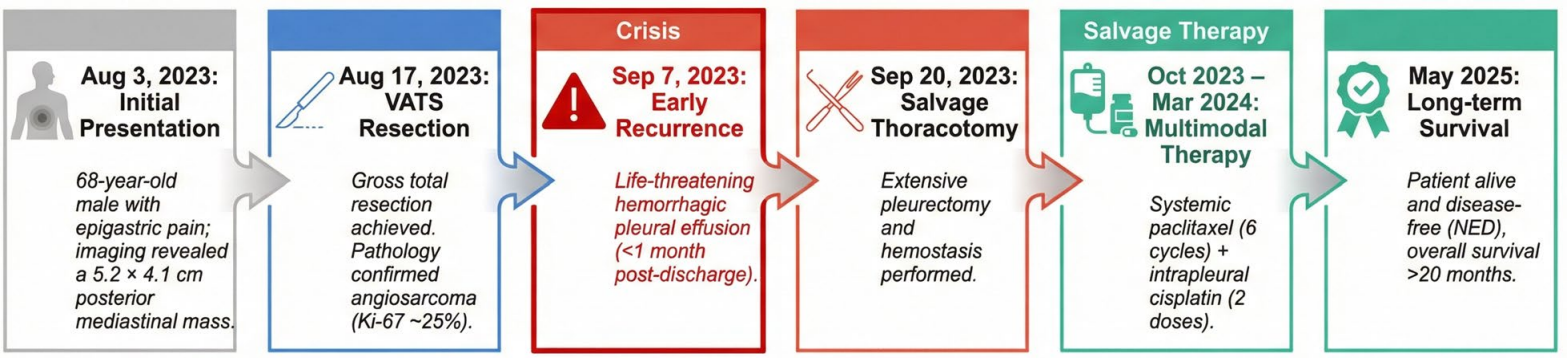
Clinical timeline. A chronological overview of the patient’s clinical course, illustrating the initial presentation, primary VATS resection, rapid recurrence, implementation of multimodal salvage therapy (systemic paclitaxel and intrapleural cisplatin), and long-term survival outcome

The patient tolerated treatment well with manageable side effects (grade 1 neutropenia and transient nausea). After 6 cycles of paclitaxel therapy, serial contrast-enhanced CT scans at 3, 6, and 12 months demonstrated Stable Disease (SD) and complete resolution of the pleural effusion. A mutual decision was made to discontinue paclitaxel to minimize cumulative toxicity and transition to a surveillance-only strategy. At the last follow-up in May 2025, the patient remained alive with no signs of disease progression more than 20 months after recurrence, maintaining an Eastern Cooperative Oncology Group performance status (ECOG PS) of 1 and good quality of life.​ The overall clinical timeline is illustrated in Fig. 5.

## Discussion

Primary mediastinal epithelioid angiosarcoma is a diagnostic and therapeutic challenge due to its rarity and nonspecific clinical presentation [4]. Our case is notable for several reasons:

First, the initial manifestation as isolated upper abdominal pain initially misdirected clinical attention toward gastrointestinal pathology, highlighting that deep thoracic tumors may refer pain to distant sites and mimic intra-abdominal pathology [5]. This underscores the importance of including mediastinal imaging in the workup of unexplained chronic abdominal complaints, especially in older adults.

Second, despite complete VATS resection—a minimally invasive approach increasingly adopted for posterior mediastinal masses [10]—the tumor recurred within **one month**, indicating highly aggressive biology. Such extreme rapid recurrence might be attributed to microscopic pleural invasion or tumor rupture during the initial VATS, which was underestimated given the benign pre-operative diagnosis. The high Ki-67 index (~ 25%) further underscored its aggressive biological behavior [6].

Third, rather than opting for palliative care or single-agent therapy, we implemented an **intensified multimodal salvage strategy** combining systemic paclitaxel and localized cisplatin perfusion. While paclitaxel has demonstrated efficacy in cutaneous and visceral angiosarcomas [7], its use in mediastinal EA is scarcely reported. Similarly, intrapleural chemotherapy is typically reserved for symptom control in malignant effusions [8], but here it likely contributed to local disease containment.

While single cases cannot be statistically compared to group data, our patient’s survival of over 20 months compares favorably to the typical prognosis of general angiosarcomas, where median survival ranges from 6 to 12 months [9]. Only a few case reports describe long-term survivors, usually following aggressive multimodal interventions [11].

The mechanism underlying the success likely involves synergistic effects: systemic paclitaxel was used to control occult systemic progression, while intrapleural cisplatin served as an acute salvage intervention to stop massive malignant bleeding, leveraging its rapid pleural penetration to achieve high local drug concentration against residual pleural micrometastases [12]. This approach aligns with emerging trends in sarcoma management emphasizing combined modality regimens tailored to individual risk profiles.

Nonetheless, several limitations exist. As a single-case study, the findings may not be broadly generalizable. Standard preoperative PET-CT and formal R0/R1 margin assessments were not performed due to the initial clinical misdiagnosis of a benign neurogenic tumor. Furthermore, we did not perform detailed molecular testing (e.g., MYC amplification) as it was unavailable at our institution, nor did we use targeted agents or immunotherapy [13]. Future studies should investigate biomarkers predictive of response to taxanes or platinum agents in EA.

## Conclusion

This case demonstrates that even in the setting of early recurrence following resection of primary posterior mediastinal epithelioid angiosarcoma, a proactive multimodal salvage strategy incorporating systemic paclitaxel and intrapleural cisplatin can lead to durable disease control and long-term survival. We advocate for heightened awareness of atypical presentations, early use of advanced imaging, and personalized, aggressive treatment approaches in managing these rare and lethal tumors.

## Data Availability

The datasets used during the current study are available from the corresponding author on reasonable request.
